# Particle radiotherapy for breast cancer

**DOI:** 10.3389/fonc.2023.1107703

**Published:** 2023-08-16

**Authors:** Hanguang Ruan, Masahiko Okamoto, Tatsuya Ohno, Yang Li, Yuan Zhou

**Affiliations:** ^1^ Department of Radiation Oncology, Gunma University, Maebashi, Japan; ^2^ Gunma University Heavy Ion Medical Center, Gunma University, Maebashi, Gunma, Japan; ^3^ Department of Radiation Oncology, Harbin Medical University Cancer Hospital, Harbin, Heilongjiang, China

**Keywords:** particle, proton, carbon ion, photon, radiotherapy, breast cancer

## Abstract

Breast cancer is the most common malignant tumor in female patients. Along with surgery, radiotherapy is one of the most commonly prescribed treatments for breast cancer. Over the past few decades, breast cancer radiotherapy technology has significantly improved. Nevertheless, related posttherapy complications should not be overlooked. Common complications include dose-related coronary toxicity, radiation pneumonia, and the risk of second primary cancer of the contralateral breast. Particle radiotherapy with protons or carbon ions is widely attracting interest as a potential competitor to conventional photon radiotherapy because of its superior physical and biological characteristics. This article summarizes the results of clinical research on proton and carbon-ion radiotherapy for treating breast cancer

## Introduction

Based on GLOBOCAN estimates of cancer incidence and mortality produced by the International Agency for Research on Cancer, breast cancer has become the most common malignant tumor in humans in 2020, surpassing lung cancer, with an estimated 2.3 million new cases representing 11.7% of all cancer cases (1). Surgery is the treatment of choice for early breast cancer. In patients who require breast-conserving and radical mastectomies, radiotherapy plays a major role in breast cancer treatment after surgery (2–4). It not only can reduce the probability of locoregional recurrence and distant metastasis, but also prolongs disease-free survival (DFS) and overall survival (OS) time (5–7). Clarke et al. reported a 5.4% decrease in 15-year breast cancer mortality with local radiotherapy (8).

The heart and lungs are the main organs at risks (OARs) in breast cancer patients receiving radiation. To ensure coverage of the planning target volume, the heart and lungs are likely to receive radiation at the same time because they are located directly posterior to the breasts. Radiation pneumonitis (RP) is one of the complications of radiotherapy for breast cancer. Its severity is closely related to the radiation dose and volume. The probability of this complication is higher in patients who need internal mammary node (IMN) irradiation (9, 10). Mehnati et al. investigated a study predicting the risk of RP and pulmonary function changes after breast cancer radiotherapy, in which V_10_ was associated with RP incidence. When V_10_ was <40% and ≥40%, the incidence of RP is 5.26% and 61.54%, respectively (11). To investigate the incidence of RP, Lee et al. identified 1, 847 women with breast cancer who received adjuvant radiotherapy. The follow-up period was 14.5 months, and the overall RP rate was 2.1%. The conclusion was that ipsilateral lung V_30_ at an equivalent dose of 2 Gy per fraction was the most significant dosimetric factor associated with RP development and showed that new RT techniques and a hypofractionation scheme significantly reduced the ipsilateral lung dose (12). There is also a linear relationship between the cardiac dose and late radiation-related cardiac toxicity morbidity, similar to radiation-induced pneumonitis. The incidence of major coronary events (MCEs) increases as the follow-up time for early breast cancer is prolonged (7, 13–15). A review has shown that the average cardiac dose was 5.4 Gy for left-sided breast irradiation compared to a dose of 3.3 Gy in cases of right-sided photon irradiation with intensity-modulated radiotherapy (IMRT) technique (16). Area et al. reported that the mean cardiac dose was 9 Gy for breast photon irradiation with IMRT, compared to 1 Gy (RBE) (relative biological effectiveness) for breast proton irradiation; the mean dose of the ipsilateral lung was 17 Gy (RBE) and 7 Gy (RBE), respectively. To reduce dose-related OARs toxicity and normal tissue damage, the development of new radiotherapeutic techniques is imperative (17).

**Figure 1:**
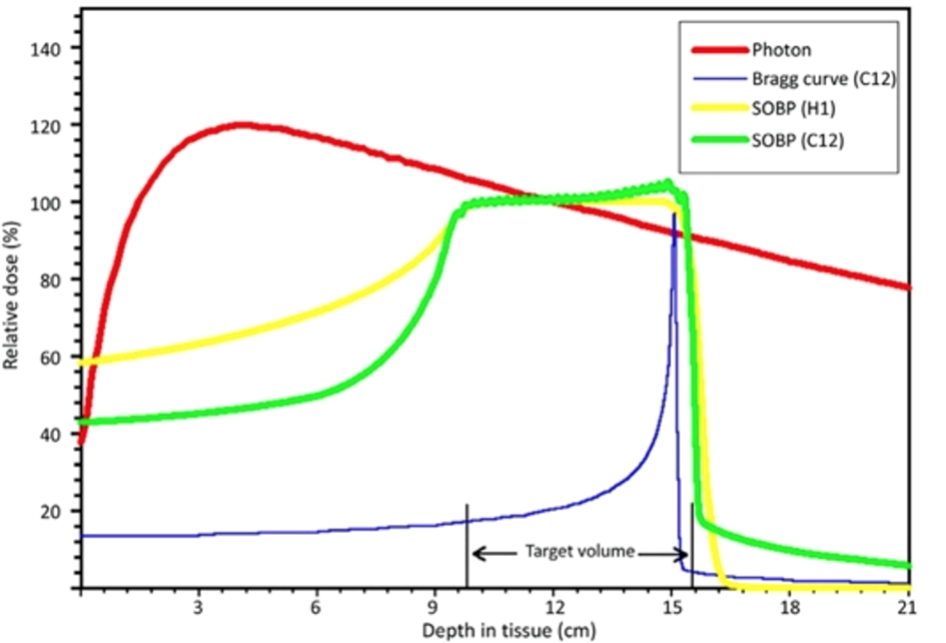
Schematic diagram of depth-dose distributions of different radiation modalities [LUKAS SCHAUB et al. (18)]

Compared to conventional photon radiotherapy, the physical characteristic feature of carbon ions and proton beams is identified as the “Bragg peak”. It is a steep and localized peak of dose that enables precise delivery of the radiation dose to the tumor target while effectively sparing normal organs and tissues (18) (**Figure 1**). Carbon-ion radiotherapy (CIRT) has obvious physical and biological advantages, including high linear energy transfer (LET) radiation. They demonstrate high-LET qualities with the Bragg peak and Low-LET behavior in the entrance channel of their trajectory. Its unique biological advantage is that higher LET radiation induces more severe DNA double-strand breaks (DSB) than lower LET radiation. In this case, many irradiated cells disable their capacity to repair the lesions after higher LET radiation, where the RBE can increase. Their advantageous biological effects have in the meantime been realized in several thousand successfully treated patients, while minor patients with breast cancer (19). However, in clinical operation, RBE depends on several factors, such as cell lines, radiation dose, fractionation, cell cycle, and oxygenation.

With a continuous improvement in understanding particle radiotherapy technology and its characteristic features by clinicians and researchers, particle radiotherapy has become an important part of radiotherapy. As of 2020, 91 facilities worldwide are fully operational; however, breast cancer is rarely treated in these facilities (13). Radiation oncologists currently focus on the use of particle radiotherapy for early breast cancer with a low risk of recurrence with or without surgery. The main research items include conventional whole-breast irradiation (WBI) with or without nodal irradiation, dose boost, post-mastectomy chest wall radiotherapy, and accelerated partial breast irradiation (APBI) using proton beams. Dosimetric evaluation of particle radiotherapy, reduction of photon radiotherapy-associated toxicities (pneumonitis, dermatitis, patchy atrophy, telangiectasias, and cardiotoxicity), improvement of cosmetic effects, and local control (LC) of breast disease are current research primary endpoints and highlights.

Particle radiotherapy for breast cancer has seen rapid growth because of improved access to proton centers worldwide. This mainly includes proton radiotherapy (PT) and CIRT. Most studies on proton and carbon ion radiation, specifically the latter, used in breast cancer have single samples, are single-center, and retrospective. Some of the published literature is concerned with metrology comparisons of the target coverage between carbon ions or proton and photon beams (20–24). Clinical radiation oncologists have encountered a bottleneck in practical applications such as radiation physics, radiobiology, and technology extension, which limits the application of particle therapy for breast cancer. Given the potential of proton and carbon ion beams and the need to clarify the benefits and challenges of proton and carbon ion beams in treating breast cancer, we have summarized clinical research outcomes from the relevant literature.

## Methods and materials

We mainly focused on clinical research on PT and CIRT for early-stage and locally advanced breast cancer. Search terms, which included “proton radiotherapy”, “carbon ion radiotherapy”, “early-stage breast cancer”, “locally advanced breast cancer”, “cardiac toxicity”, “radiation pneumonitis”, “clinical efficacy”, “clinical outcome”, “disease-free survival”, “local control” “overall survival” were used to search original articles, except reviews, published by English in PubMed/Medline, Web of Science, and Cochrane Library. The publication date was updated to February 2022.

The recruitment literatures were assessed and analyzed. The evaluation included treatment clinical efficacy and safety. The outcomes in these literatures contained short-term effects of disease LC, and DFS and OS. Acute and late adverse events (AEs), and cosmetic results also were reported.

## Results

### Past and present in proton and carbon ion radiotherapy

Proton and carbon ion beam therapies are the main components of particle radiotherapy. Professor M. Oliphant designed a proton accelerator in 1952. Proton beam therapy has evolved since its first use in 1954 at the Lawrence Berkeley Laboratory. In 1958, the same group reported the first clinical data using accelerated protons: 26 patients with advanced breast cancer received 340 MeV proton beam therapy to the pituitary gland for hormone suppression in a palliative setting. Tsukuba and Loma Linda University conducted the initial clinical implementation of PT in breast cancer patients. Over the next two decades, a number of PT projects have been developed, and their results have been reported. Heavy ions, including helium, carbon, and nitrogen ions, began to develop in the 1970s (18). In 1993, the Japanese government built the world’s first heavy ion medical accelerator in Chiba (HIMAC) at the National Institute of Radiological Science (NIRS) in Chiba Prefecture (25, 26). The first case report was published by NIRS in 2014, which discussed the effectiveness of carbon ion beam for the treatment of stage I breast cancer without surgery (27).

**Figure 2:**
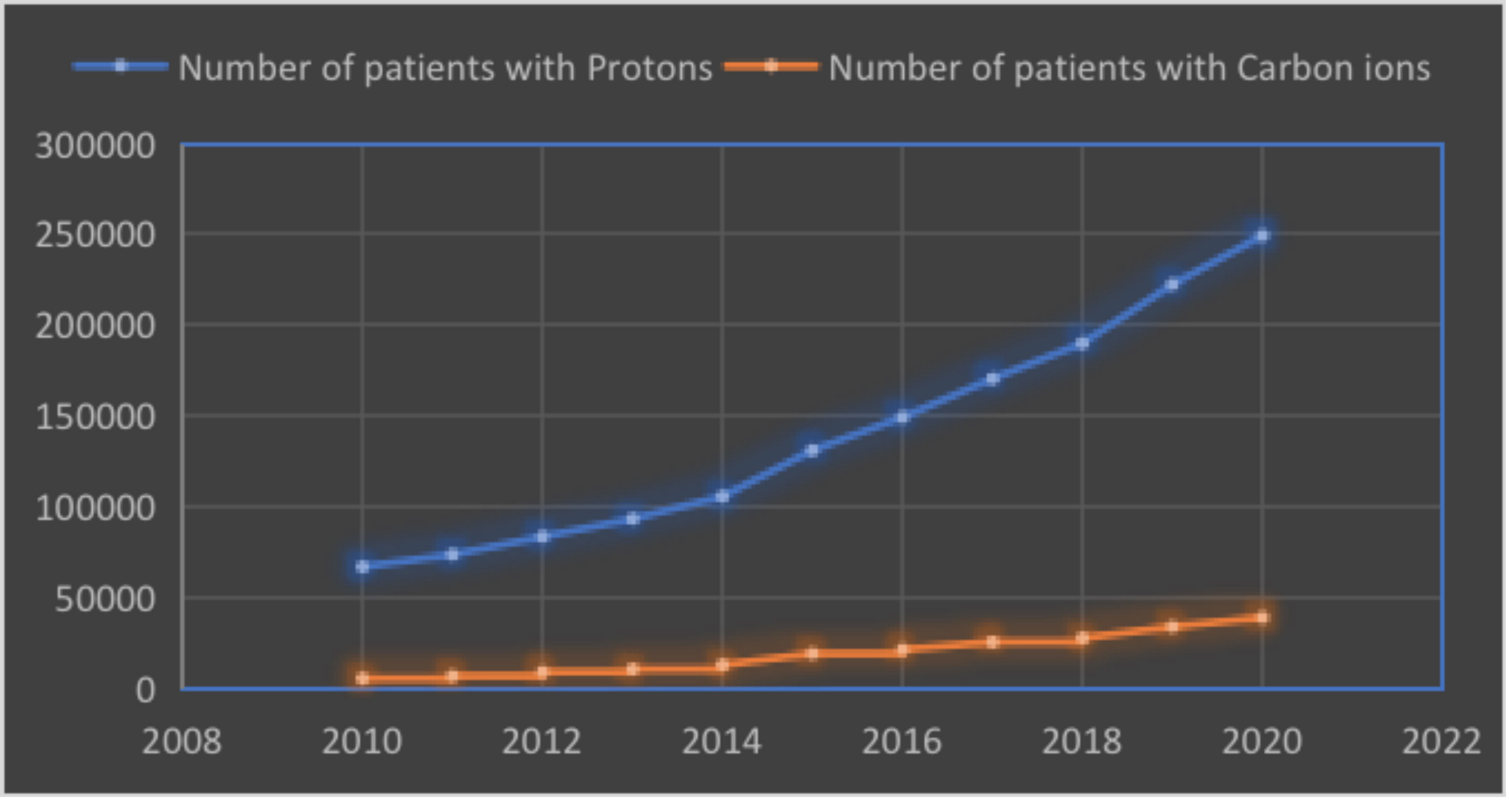
The number of patients with protons and carbon ions beams (collected by the PTCOG, end of 2020).

The physical advantages of proton and carbon ion beam medical accelerators are mainly reflected in the “Bragg peak” distribution in human tissue. Carbon ions and protons have similar physical properties; however, compared to proton beams, carbon ions tend to require more energy at the same tumor depth. Therefore, to conform to carbon ion therapy requirements, larger accelerators and beam delivery systems were required, according to particle therapy patient statistics (end of 2020) and data collected by the Particle Therapy Co-Operative Group (PTCOG) (www.ptcog.ch). The number of patients who received proton and carbon ion beams was 249,297 and 39,210, respectively. The cases of proton and carbon ion therapies in the last decade are shown in (**Figure 2**).

### Clinical outcomes of proton beam therapy

#### Radiation-related cardiotoxicity

Radiation therapy is an important component of breast cancer treatment in patients with postoperative breast cancer. However, radiation-related cardiotoxicity is a negative prognostic factor for breast cancer with irradiation. Previous studies have indicated that there is a direct relationship between the cardiac dose and cardiotoxicity (9, 28). To reduce the cardiac irradiation dose, some techniques such as IMRT and deep inspiration breath-hold (DIBH) have also been implemented in photon therapy. Michał. Falco reported that the mean heart dose (MHD) values in the patients treated with DIBH were significantly lower than in patients treated with non-gated free-breathing (FB) (2.1 vs 3.48 Gy) and gated FB (3.28 Gy) (29). Bruno. Speleers conducted a study that investigated the dosimetric effect of DIBH on the heart in both photon and proton plans for the treatment of whole breast (WB) and LN (including the MI chain). The results showed that DIBH significantly decreased dose to heart for photon and proton radiotherapy (30).

**Figure 3:**
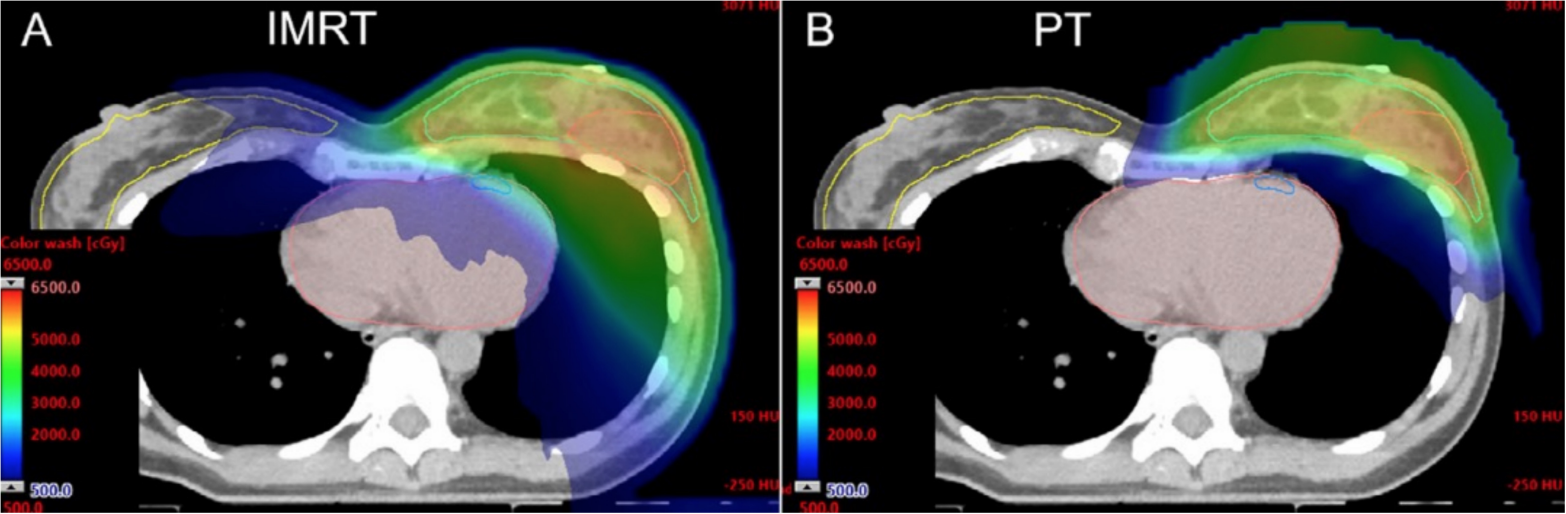
Computed tomography (CT) dose color wash from an IMRT plan **(A)** and pencil-beam scanning PT plan **(B)** [Mutter et al. (13)].

On the other hand, based on the physical characteristics of proton radiation, PT can optimize irradiation dose of heart and surrounding tissues compared with conventional photon radiotherapy, especially when the internal mammary chain region receives radiation therapy (13). The Particle Therapy Cooperative Group Breast Cancer Subcommittee (PTCGBCS) reported that PT reduces the dose to the heart compared with 3-dimensional conformal radiation therapy (3DCRT) and IMRT from computed tomography (CT) therapy plan (13) (**Figure 3**). Verma et al. (31) conducted a study in which the median PT whole-breast dose was 50.4 Gy (RBE), with a subsequent boost as clinically indicated (median 10 Gy (RBE]). The results showed that the ipsilateral lung constraints were V_20_ ≤ 21% and heart V_5_ ≤ 50% and ≤ 40% for left- and right-sided cases, respectively. Sigole et al. (32) conducted a study and showed that the DIBH technique can further reduce cardiac radiation dose using proton radiation. It was shown that the maximum dose to the heart was 3.8 Gy (RBE).

Since systematic treatment regimens for breast cancer have been optimized, the OS rate of patients with breast cancer has substantially improvement. Meanwhile, long-term dose-related cardiotoxicity after radiotherapy has attracted more attention (14, 33–35). Incidental exposure of the heart to irradiation for breast cancer increases the rate of MCEs by 7.4% per gray. The risk may be higher for patients with underlying cardiovascular disease and patients who smoke (36, 37). Limiting the cardiac radiation dose from breast irradiation and reducing late radiation-related cardiac morbidity for breast cancer are supported by the currently available literature.

#### The profile of clinical outcomes

The currently published literature on the application of protons in breast cancer lacks multicenter, randomized, large-scale phase III clinical studies. The reason for this is the limited number of cancer centers conducting PT and the lower cost-benefit ratio of PT. Most of the literature on PT in early and locally advanced breast cancer (LABC) is a single-center, small-sample exploratory study. They have focused on the coverage of the irradiation target area, the tumor LC rate, the acute and late AEs of the treatment, and the occurrence of cardiotoxicity and dose-related RP. The target population of the study was mainly early breast-sparing APBI and patients with locally advanced breast cancer requiring internal mammary node radiation (38–43) (**Table 1**).

**Table 1:** Clinical outcomes of proton beams therapy.

Authors	Years /Patients	Following Time	Target Dose and Modality Technique	Target Area	Disease Control	Adverse Events	
						Acute	Late
Vivek Verma et al. (31)	2011–2016, 91 patients Left: 56 Right: 33 Bilateral: 2 LABC	Median follow-up: 15.5 months	Median 50.4 Gy (RBE) PT: three-dimensional uniform scanning Pencil beam scanning	Breast Chest wall	12 patients experienced disease failure (10 recurrence)	Dermatitis: Grades 1, 2 and 3 occurred in 23%, 72% and 5% Esophagitis: Grade 2 and 1 occurred in 33% and 31% respectively	Rib fracture: Two patients (2%)
John J. Cuaron et al. (43)	2013-2014, 30 patients LABC	Median follow-up: 9.3 months	Median 50.4 Gy (RBE) PT: postoperative	Breast Chest wall Regional lymph nodes including IMN	–	Dermatitis: Grade 2 in 20 patients (71.4%) and 8 (28.6%) experiencing moist desquamation Esophagitis: Grade 2 in 28.6% (8 patients)	Reconstructive complications: Grade 3 in 1 patient
Sigole`ne et al. (32)	2003-2006 98 patients EBC	Median follow-up: 82.5 months;	Median 32 Gy (RBE) PT or Photon APBI	Breast Chest wall	7 years LF: 11%PP, 4% EB or P/E, NSS	Skin color change	Overall cosmesis: As fair in 44% of PBT patients
Bush et al. (41)	2003-2006 100 patients EBC NCT00614172	Median follow-up: 60 months;	Median 40 Gy (RBE) PT	Breast Chest wall	5 years ipsilateral breast tumor RFS: 97% DFS: 94%, OS: 95%	Moderate radiation dermatitis: Graded as 1 or 2 in 62%	Telangiectasia: Grade 1in 7 cases; good to excellent result: of 90% for 5 years
Ji Hyun Chang et al. (42)	2007-2009 30 patients EBC	Median follow-up: 59 months	Median 30 Gy (RBE) PT	Breast Chest wall	3 years LF and DF 0%	Wet desquamation: Grade 1 in 1 patient had at 2 months	Cosmetic good or excellent: Noted in 83% and 80% respectively
Jimenez et al. (40)	2011-2016 69 patients EBC	Median follow-up: 55 months	Median 49.7 Gy (RBE) PT	Breast Chest wall	5 years LF: 1.5% and OS: 91%	Grade 2 RF in 1 patient	The unplanned surgical re-intervention rate at 5 years was 33%

LABC, locally advanced breast cancer; EBC, early breast cancer; PT, proton radiotherapy; CIRT, carbon ion radiotherapy; IMN, internal mammary lymph nodes; APBI, Accelerated Partial Breast Irradiation; PP, passively scattered proton therapy; EB, photon external beam radiotherapy; P/E, photons with electrons; NSS, not statistically significant; RFS, recurrence-free survival; DFS, disease free survival; OS, disease-free survival; LF, local failure; DF, distant failure; RF, radiation pneumonitis; IMN, internal mammary nodes.

#### Accelerated partial breast irradiation

Sigole`ne et al. (32) published an article, in which they detailed how they recruited 98 patients of early-stage breast cancer after breast-conserving surgery (BCS) to be treated with proton and photon beam therapy. This study was a first-dose cohort of a phase I APBI trial conducted at the Massachusetts General Hospital (MGH), which included a small number of patients treated with PT. The long-term results showed that PT had a lower ipsilateral lung mean and maximum dose and greater non-target breast sparing. However, outcomes of the 7-year incidence of local failure (LF) or patient-reported cosmetics showed no significant difference between the PT and photon groups. A phase 2 trial of 30 patients was conducted by the National Cancer Center in Korea. This study reported that the long-term results of physician-assessed cosmetic outcomes were good or excellent in 69% at 3 years, but increased to 89% for patients treated with two fields. 3 years LF and distant failure (DF) rates were 0% (42).

Bush et al. (41) reported a phase 2 trial using proton beam radiation to deliver APBI to patients with early-stage breast cancer. The eligibility criterion was that the tumor should be an invasive cancer with a maximal dimension of 3 cm. The patients underwent partial mastectomies with negative margins, and the axillary lymph nodes were negative on sampling. The research sample comprised 100 enrolled and treated patients. The selected dose of partial breast irradiation (PBI) therapy was 40 Gy (RBE) administered in 10 fractions. The results showed that the 5-years ipsilateral breast tumor RFS was 97%, DFS was 94%, and OS was 95%. Regarding AEs, there were no cases of grade 3 or higher acute skin reaction, but the reported late skin reaction included seven cases of grade 1 telangiectasia. Pasalic et al. reported a prospective phase 2 trial clinical outcome at a planned interim analysis treated with proton APBI to 34 Gy (RBE) in 3.4 Gy (RBE) twice-daily fractions after breast-conserving surgery for 100 patients with pTis or pT1-2 N0 (≤3 cm) breast cancer status. The median follow-up period was 24 months (range:12-43). The LC and OS were 100% at 12 and 24 months, respectively. There were no acute or late toxicities of grade 3 or higher, and no patients developed fat necrosis, fibrosis, infection, or breast shrinkage. The most common toxicity was grade 1 or 2 late breast-skin telangiectasia. The incidence rate was 17%. The author indicated that the radiation coverage volume (>610 cm^3^) and the number of beams were the strongest factors associated with developing telangiectasia, based on multivariate Cox regression analysis. Dosimetric analysis revealed that there was a lower mean left-sided heart and ipsilateral lung dose of 2 cGy (range:0.2-75) and 19 cGy (range:0.2-164), respectively (44).

The above studies are typical representative studies on early breast cancer with proton APBI, which demonstrate exceptional heart- and lung-sparing effects, a high LC rate, favorable cosmetic outcomes, high patient satisfaction, and minimal treatment time. Some studies have shown that it can reduce the impact of time off work (24). However, there is still some arguing about less fibrosis, more incidents of telangiectasias using protons compared to photons, and the dosimetric advantages whether will lead to reduced long term cardiac and lung toxicity. In addition, unfortunately, rib fractures appear to be more common with protons. Owing to the present arguing, further large-scale clinical studies will answer the aforementioned questions.

#### Regional node irradiation

Regional node irradiation (RNI) is an important component of breast cancer radiotherapy for LABC. RNI includes the axillary, supraclavicular (SC), and IMN. Owing to RNI, especially the addition of radiotherapy to the IMN, the radiation dose to the heart and ipsilateral lung will be greatly increased, especially in left breast cancers. PT has the potential to reduce radiation dose to the heart, lung and intrinsic muscles, and which also may reduce the incidence of dose-related toxicity, such as shoulder disability, chest wall pain, and upper-limb lymphedema.

A prospective trial result was published with long-term follow-up of patients with breast cancer undergoing RNI using a proton beam. 69 patients underwent passive scattering (PS) or pencil-beam scanning (PBS) PT, 93% underwent mastectomy, and 7% underwent a lumpectomy. The outcome of the trial was that the mean heart dose, left-side anterior descending artery (LAD) max dose, and ipsilateral lung V_20_ Gy (RBE) values were 0.5 Gy (RBE), 4.7 Gy (RBE), and 14.5%, respectively. In this case, 5-years LC was 98.5%, and the OS was 91% (40). A clinical study conducted by the Memorial Sloan Kettering (MSK) Cancer Center showed that in 42 patients who received PT with RNI with a median follow-up of 35 months, no grade 3 or greater acute toxicities were noted, and only 1 (2%) had grade 3 late complications. The results showed that the 3-years DFS was 96.3%, metastasis-free survival (MFS) was 84.1%, OS was 97.2%, and LC was 97.6% (45).

A study focused on the selection criteria for early breast cancer patients using proton beams in the Danish Breast Cancer Group (DBCG) proton trial, a random phase III trial strategy. The endpoint of the study was to determine the estimated heart and lung doses when the target coverage was not compromised in consecutive patients. The recruited 179 breast cancer patients had already been treated with loco-regional IMN radiotherapy. The planning techniques included 3DCRT and volumetric modulated arc therapy (VMAT), hybrid planning techniques (combination of 3DCRT and VMAT), and IMRT. This trial concluded that patients with MHD of 4 Gy and/or ipsilateral lung V_17_/V_20_> 37% were candidates for the random DBCG Proton Trial (23, 46). No data on therapeutic efficacy and AEs were reported in this study and only some reports on dosimetric comparisons of target coverage and OARs with different therapy techniques. Even so, this retrospective study estimated heart and lung doses in breast cancer patients receiving locoregional IMN radiotherapy. The results of this study showed that 60% of the treatment plans had already met the delineation requirements and DBCG target coverage constraints. According to the inclusion criteria of the DBCG radiotherapy plan, the heart and lung radiation doses could be lower than that shown in the actual clinical data, as was the incidence of late cardiopulmonary toxicity.

This retrospective clinical study implies that radiation oncologists should consider significant clinical considerations when planning therapy. For instance, physicians need to consider whether patients present with any cardiopulmonary diseases, smoking habits, and combined systemic chemotherapy or targeted therapy drugs. The actual exposure and risk of OARs (affecting the heart and lungs) may often differ from what is estimated in theoretical data according to the patient’s physical condition (9, 47). This also provides further encouragement for radiation oncologists to be conservative when making treatment planning. On the other hand, it is worth noting that clinical researches of LABC treatment with PT have been single-center, small-sample studies focused on dose assessment and reports of AEs after RNI treatment.

A Radiotherapy Comparative Effectiveness (RadComp) Consortium Trial (NCT02603341) is an ongoing large-scale, multicenter pragmatic random clinical trial for non-metastatic breast cancer. All patients receive breast/chest wall and comprehensive nodal radiation therapy including IMN treatment. The objective of study is to evaluate whether the differences between and photon radiotherapy and PT cardiac radiation dose distributions lead to meaningful reductions in cardiac morbidity and mortality after treatment (48). According to the results of DBCG-IMN trial, IMN radiotherapy caused a significant increase in dose to the heart and lung in spite of gaining OS from IMN radiotherapy (49). In this context, another ongoing study, the DBCG proton trial (NCT04291378) is being conducted by Danish Breast Cancer Cooperative Group, randomizing patients between standard photon radiotherapy versus experimental PT for early breast cancer. The primary endpoint is 10-year risk of radiation associated ischemic and valvular heart disease. The final outcomes about the potential cardiac benefit in proton are deserving of expectation.

### Clinical outcomes of carbon ion radiotherapy

Compared to protons, carbon-ion beams have physical and biological unique properties. However, CIRT is not as widely used as PT in clinical practice. There are the causes as following. First, CIRT is suitable for intact breast cancers, and applicable for early stage tumors requiring localized treatment. This is because CIRT has a high LET property, which makes it more suitable for irradiation of localized tumors, but is not fit for prophylactic irradiation of large areas because it also causes more damage to the normal tissue in the irradiated area. Second, the application for CIRT is nascent. By 2020, there were only five countries with carbon ion therapy facilities and 12 facilities worldwide, with cost and resource barriers being the main causes, especially in developing countries. Third, there are still many uncertain factors surrounding the application of carbon ion beams to breast cancer, including prescription dose, irradiation segmentation, and combination drug efficiency (50, 51).

The first case report of CIRT applied to the breast was performed in NIRS in Japan. It showed that the first observed patient was a 50-years old without clinical symptoms presenting with an abnormality on screening mammography and left early-stage breast cancer. The breast tumor was not surgically resected. The description dose was a total of 52.8 Gy (RBE) in four fractions of 13.2 Gy (RBE). With the follow-up three months after CIRT, the outcome showed a reduction in tumor size, which, however, did not completely disappear. Acute AEs were reported as only grade 1 adverse skin reactions (27). Regrettably, the researcher failed to report DFS and OS rates after CIRT during the follow-up period. The clinical outcomes of CIRT are shown in **Table 2** (21, 27).

**Table 2:** Clinical outcomes of carbon ions beams therapy.

Authors	Years /Patients	Following Time	Target Dose and Modality Technique	Target Area	Disease Control	Adverse Events	
						Acute	Late
Kumiko Karasawa et al. (21)	2013-2015, 21 patients EBC Left: 3 Right: 11 (7 patients beyond recruitment criteria)	Median follow-up: 61 and 37~ 48 months	3 case: 52.8 Gy (RBE) 11 case: 60 Gy (RBE) Beyond recruitment criteria: 3 case: 48 Gy (RBE) 3 case: 52.8 Gy (RBE) 1 case: 60 Gy (RBE) CIRT: respiratory gating	Breast Chest wall	CR:14 PR:2 SD:5 13 case no recurrence 1 case: Local + Axillary lymph node recurrence	Skin reaction: Grade 1 in 14patients	Good cosmetic: Not any late adverse reaction
Hiroko Akamatsu et al. (27)	2013 Left: 1 patient EBC	Follow-up: 3 months	Dose 52.8 Gy (RBE) CIRT: first experiment for breast cancer	Breast Chest wall	Not completely disappear after 3 months	Skin adverse reaction: Grade 1	–

EBC, early breast cancer; CIRT, carbon ion radiotherapy.

**Figure 4:**
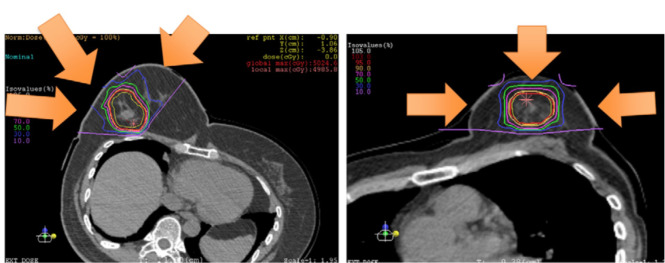
Portal setting and dose distribution of the patients [Karasawa et al. (39)]. Arrow means the deliver beams direction.

Karasawa et al. (39), conducted a phase I clinical does escalation trial of CIRT for 7 patients with low risk stage I breast cancer. It was planned that patients would undergo primary tumor excision and sentinel lymph node biopsy for pathological evaluation three months after CIRT. Three patients received 48 Gy (RBE), three patients received 52.8 Gy (RBE), and one patient received 60 Gy (RBE). A dose distribution image of the CIRT is showed (**Figure 4**). The primary endpoint was acute AEs, and the secondary endpoint was tumor control rate. The clinical outcomes were that two patients had partial response (PR) and 5 patients showed stable disease (SD). Three months after CIRT, one patient had a complete response (CR), and one patient had SD. Following resection, all the tumors had negative surgical margins. None of the patients had other AEs, except for four patients who experienced grade 1 acute skin reactions. Similarly, 14 patients received CIRT with a dose escalation (21). The results were one patent had a CR and 13 had a PR at 3 months after CIRT. At 6 months point in time, four CR, nine PR, and one progressive disease (PD) were observed, and at 24 months point in time, 13 CR was noted. Acute AEs included grade one skin reaction in 10 patients. However, there is limited literature on the use of CIRT in the treatment of breast cancer, and most cases are related to early breast cancer. This study was homogeneous and exploratory and included dose escalation, target coverage, shorter survival time, and observation of AEs.

## Discussion

Breast cancer is the most common malignancy worldwide and remains the leading malignant tumor in women. Radiotherapy plays an important role in the treatment of breast cancer. The irradiation associated research paradigm is not only aimed at increasing cancer survivorship, but also on minimizing radiation toxicities that can compromise the quality of life (QoL) of early breast cancer patients, especially late complications such as cardiovascular disease, arm and shoulder function, and second primary cancer. Thus, dosimetric benefits for critical organs and normal tissues have become a major focus of attention for radiation oncologists, as observed for other oncological scenarios (52, 53).

### Particle property profiles of particle radiotherapy

PT and CIRT exhibit a narrow area of high-energy deposition with a sharp dose fall-off (51, 54). They are better at sparing normal tissue or OARs, nearby a radiation target, than conventional radiation. Even so, some aspects of particle therapy for breast cancer still need to be considered.

### Physical advantage and associated toxicities

The EORTC 22922 and MA.20 trials showed that radiation therapy target coverage of the IMN improved OS and DFS rates in patients with high-risk breast cancer. However, IMN irradiation is associated with increased cardiac and ipsilateral lung exposure to radiation, thereby increasing the potential AEs of therapy, particularly in left-sided breast cancer (55, 56). Cardiovascular events after a few decades have been translated by excess and unintended irradiation doses to the OARs. To reduce these risks, target dosimetric optimization is a potentially effective means, including tumor target conformal coverage and low-dose irradiation in the surrounding normal tissue. Particle therapy can precisely optimize this radiotherapy disadvantage owing to its sharp dose gradient. However, the therapeutic targets of breast cancer change with respiratory movement result in some planned therapeutic targets may receive incomplete coverage due to respiratory movement, or the dose received in the target area deviates from prescription. Consequently, techniques for reducing the impact of respiratory movements on the target area are important for precise treatment.

As mentioned in the results section, to reduce the irradiation dose to cardiac, lung, and other normal tissues such as the intrinsic muscles of the shoulder and chest, some techniques such as DIBH and EIG have also been implemented in photon therapy. However, no universal consensus or prospective studies have confirmed the viewpoints and benefits of combining techniques with particle therapy (57). In addition to the impact of respiratory movement on the target, the daily radiation positioning deviation should be considered, although some radiation oncologists suggest that daily indoor CT scans, repositioning, and repeat planning of radiotherapy can reduce the severity of the deviation (58). However, there is no consensus on how much of the daily target deviation is acceptable, even though most radiation oncologists currently consider the deviation of conventional photon radiotherapy to be within the acceptable range of ± 3 mm (59, 60). How this is not necessarily the case for protons, where small shifts may cause larger deviations in dose distribution.

### Biological advantage and challenges

Unique biological properties are the central advantage of particle therapy. The universal consensus is that the average radiobiological equivalent dose of particles relative to photon beams is approximately 1.1. In contrast, the RBE of carbon ions is not a constant value, but a function of the position within the treatment beam (61, 62). The most relevant challenges of proton therapy are the uncertainties related to the different beam penetration ranges in tissues and changes in the magnitude of the RBE along the beam path. The proton therapy prescription and constraints are based on dose parameters and dose-response relationships derived by photon therapy. Different dose responses may be achieved with different therapeutic modalities. Some studies have suggested that dose fraction, tissue and cell type, and oxygenation are the main factors that influence the dose-response after radiotherapy (63). The actual RBE dose date may change along the beam path. At the beam tail, the proximity of the Bragg peak increases significantly. This phenomenon is associated with the increased LET of the proton beam at the distal edge. The extended beam range of proton therapy is usually 2-4 mm with an increase in RBE at the beam tail (43, 64). In practice, the biological dose received by normal tissue may be higher than the prescribed effective dose owing to the RBE heterogeneity and coverage targeting, causing unexpected damage to the surrounding normal tissue, such as radiation pneumonia and rib fractures. The extended range and increase in RBE at the beam tail may also land increased dose at the LAD, which could lead to worsening heart dose (65). This is unacceptable for young, early-stage, and high-longevity patients with breast cancer.

### Particle radiotherapy and systematic therapy

It is worth noting that evidence in terms of efficacy and AEs combinations for adjunctive antitumor drug and particle synchronous therapy for early breast cancer is lacking. Most of these effects are based on changes in the internal environment and drug interactions caused by radiotherapy, such as adjuvant enhancement of capecitabine or anthracyclines in triple-negative breast cancer (TNBC) and CDK4/6 inhibitors in luminal subtype patients with a high risk of recurrence after surgery. Trastuzumab and Pertuzumab are commonly recommended for use in patients with breast cancer with HER2 gene overexpression for one year (66–70). These drugs carry a toxic risk for heart failure and interstitial pneumonia (11, 34, 71). There is little clinical evidence to confirm whether this type of AEs increases in patients treated with particle radiotherapy, as it is difficult to conduct large clinical studies because of the few particle therapy facilities currently in operation. Even so, the current clinical research results on particle therapy for early breast cancer offer some prospects for clinical radiation oncologists.

### The prospect of particle radiotherapy

Though PT and CIRT have clear physical and biological advantages over conventional photon radiotherapy. According to clinical application, particle therapy is not as widely used as photon for early breast cancer. Suboptimal patient selection is a potential factor. The majority of early breast cancer patients who receive particle therapy as radical treatment are at low risk of recurrence or cannot tolerate surgery because of physical reasons such as cardiopulmonary failure (72). Further, technical challenges of particle therapy also remain concern. At the robustness and end of range effects, proton beams have the characteristics of variations in increased LET and RBE at the distal edge of them, which may lead to an increase in toxicity (13, 57).

In addition to radiotherapy, the definitive treatment approaches for early breast cancer also include surgery and cryoablation. However, here are some potential drawbacks of surgery, such as limited preservation of breast tissue, surgical risks and complications, and potential lymphedema (73, 74). Meanwhile, cryoablation has the unique properties, including limited applicability, incomplete assessment of lymph nodes, and limited long-term data for application (75). Despite there are some variations in particle therapy for breast cancer such as robustness calculations. The ballistic and radiobiological properties of particle beam make it a potential treatment option for radioresistant breast cancer subtypes. The suitable and rigorous research is imperative to confirm this.

## Conclusions

Particle therapy has developed rapidly in recent years as it has the potential to be a powerful tool in the treatment of malignant tumors. Whether the advantages of particle therapy are over conventional photon and other approaches in these patient demographics, and particle therapy how to better integrate into multidisciplinary treatment system for early and locally advanced breast cancer. Large, multicenter, phase III clinical studies are ongoing to answer these questions.

## Author contributions

HR wrote the paper. HR, YL and YZ conceived the design of review and date collection. TO and MO reviewed drafts of the manuscript, and approved the final draft. All authors were involved in reading the papers, and unanimously agreed to the manuscript.

## References

[1] Sung H Ferlay J Siegel RL Laversanne M Soerjomataram I Jemal A. Global cancer statistics 2020: GLOBOCAN estimates of incidence and mortality worldwide for 36 cancers in 185 countries. CA Cancer J Clin (2021) 71(3):209–49. doi: 10.3322/caac.21660 33538338

[2] Overgaard M Nielsen HM Tramm T Højris I Grantzau TL Alsner J. Postmastectomy radiotherapy in high-risk breast cancer patients given adjuvant systemic therapy. A 30-year long-term report from the danish breast cancer cooperative group DBCG 82bc trial. Radiother Oncol (2022) 170:4–13. doi: 10.1016/j.radonc.2022.03.008 35288227

[3] Meattini I Becherini C Boersma L Kaidar-Person O Marta GN Montero A. European Society for Radiotherapy and Oncology Advisory Committee in Radiation Oncology Practice consensus recommendations on patient selection and dose and fractionation for external beam radiotherapy in early breast cancer. Lancet Oncol (2022) 23(1):e21–31. doi: 10.1016/S1470-2045(21)00539-8 34973228

[4] Hennequin C Belkacémi Y Bourgier C Cowen D Cutuli B Fourquet A. Radiotherapy of breast cancer. Cancer Radiother (2022) 26(1-2):221–30. doi: 10.1016/j.canrad.2021.11.013 34955414

[5] Xu FF Cao L Xu C Cai G Wang SB Qi WX. Practical model to optimize the strategy of adjuvant postmastectomy radiotherapy in T1-2N1 breast cancer with modern systemic therapy. Front Oncol (2022) 12:789198. doi: 10.3389/fonc.2022.789198 35280719 PMC8908314

[6] Luz F Marinho EDC Nascimento CP Marques LA Duarte MBO Delfino PFR. The effectiveness of radiotherapy in preventing disease recurrence after breast cancer surgery. Surg Oncol (2022) 41:101709. doi: 10.1016/j.suronc.2022.101709 35124329

[7] Kao YS. Comment to "Long term results of a phase II trial of hypofractionated adjuvant radiotherapy for early-stage breast cancer with volumetric modulated arc therapy and simultaneous integrated boost". Radiother Oncol (2022) 166:100. doi: 10.1016/j.radonc.2021.10.024 34856289

[8] Clarke M Collins R Darby S Davies C Elphinstone P Evans V. Effects of radiotherapy and of differences in the extent of surgery for early breast cancer on local recurrence and 15-year survival: an overview of the randomised trials. Lancet (2005) 366(9503):2087–106. doi: 10.1016/S0140-6736(05)67887-7 16360786

[9] Haussmann J Corradini S Nestle-Kraemling C Bolke E Njanang FJD Tamaskovics B. et al: Recent advances in radiotherapy of breast cancer. Radiat Oncol (2020) 15(1):71. doi: 10.1097/00001703-200502000-00006 32228654 PMC7106718

[10] Vaidya JS Bulsara M Wenz F Tobias JS Joseph D Baum M. Targeted radiotherapy for early breast cancer. Lancet (2018) 391(10115):26–7. doi: 10.1016/S0140-6736(17)33316-0 29323648

[11] Mehnati P Ghorbanipoor M Mohammadzadeh M Nasiri Motlagh B Mesbahi A. Predicting the risk of radiation pneumonitis and pulmonary function changes after breast cancer radiotherapy. J BioMed Phys Eng (2021) 11(4):459–64. doi: 10.31661/jbpe.v0i0.1079 PMC8385223 34458193

[12] Lee BM Chang JS Kim SY Keum KC Suh CO Kim YB. Hypofractionated radiotherapy dose scheme and application of new techniques are associated to a lower incidence of radiation pneumonitis in breast cancer patients. Front Oncol (2020) 10:124. doi: 10.3389/fonc.2020.00124 32117771 PMC7026386

[13] Mutter RW Choi JI Jimenez RB Kirova YM Fagundes M Haffty BG. Proton therapy for breast cancer: A consensus statement from the particle therapy cooperative group breast cancer subcommittee. Int J Radiat Oncol Biol Phys (2021) 111(2):337–59. doi: 10.1016/j.ijrobp.2021.05.110 PMC8416711 34048815

[14] Zareie B Rasouli MA Poorolajal J. Risk of primary lung cancer after breast cancer radiotherapy: a systematic review and meta-analysis. Breast Cancer (2022) 29(2):361–7. doi: 10.1007/s12282-021-01318-w 35088288

[15] Mulliez T Miedema G Van Parijs H Hottat N Vassilieff M Gillet E. Pre-OPerative accelerated radiotherapy for early stage breast cancer patients (POPART): a feasibility study. Radiother Oncol (2022) 174:168–9. doi: 10.1016/j.radonc.2022.02.033 35257850

[16] Shah C Badiyan S Berry S Khan AJ Goyal S Schulte K. Cardiac dose sparing and avoidance techniques in breast cancer radiotherapy. Radiother Oncol (2014) 112(1):9–16. doi: 10.1016/j.radonc.2014.04.009 24813095

[17] Ares C Khan S Macartain AM Heuberger J Goitein G Gruber G. Postoperative proton radiotherapy for localized and locoregional breast cancer: potential for clinically relevant improvements? Int J Radiat Oncol Biol Phys (2010) 76(3):685–97. doi: 10.1016/j.ijrobp.2009.02.062 19615828

[18] Schaub L Harrabi SB Debus J. Particle therapy in the future of precision therapy. Br J Radiol (2020) 93(1114):20200183. doi: 10.1259/bjr.20200183 32795176 PMC7548373

[19] Endo M. Creation, evolution, and future challenges of ion beam therapy from a medical physicist's viewpoint (part 1). Introduction and Chapter 1. accelerator and beam delivery system. Radiol Phys Technol (2022) 15(4):271–90. doi: 10.1007/s12194-022-00681-3 36348146

[20] Malouff TD Mahajan A Krishnan S Beltran C Seneviratne DS Trifiletti DM. Carbon ion therapy: A modern review of an emerging technology. Front Oncol (2020) 10:82. doi: 10.3389/fonc.2020.00082 32117737 PMC7010911

[21] Karasawa K Omatsu T Shiba S Irie D Wakatsuki M Fukuda S. A clinical study of curative partial breast irradiation for stage I breast cancer using carbon ion radiotherapy. Radiat Oncol (2020) 15(1):265. doi: 10.1186/s13014-020-01713-1 33187529 PMC7666457

[22] Kammerer E Guevelou JL Chaikh A Danhier S Geffrelot J Levy C. Proton therapy for locally advanced breast cancer: A systematic review of the literature. Cancer Treat Rev (2018) 63:19–27. doi: 10.1016/j.ctrv.2017.11.006 29197746

[23] Fuglsang Jensen M Stick LB Høyer M Kronborg CJS Lorenzen EL Mortensen HR. et al: Proton therapy for early breast cancer patients in the DBCG proton trial: planning, adaptation, and clinical experience from the first 43 patients. Acta Oncol (2022) 61(2):223–30. doi: 10.1080/0284186X.2021.1986229 34632922

[24] DeCesaris CM Pollock A Zhang B Poirier Y Kowalski E Paulosky K. Assessing the need for adjusted organ-at-risk planning goals for patients undergoing adjuvant radiation therapy for locally advanced breast cancer with proton radiation. Pract Radiat Oncol (2021) 11(2):108–18. doi: 10.1016/j.prro.2020.09.003 33109494

[25] Li Y Li X Yang J Wang S Tang M Xia J. Flourish of proton and carbon ion radiotherapy in China. Front Oncol (2022) 12:819905. doi: 10.3389/fonc.2022.819905 35237518 PMC8882681

[26] Mohamad O Yamada S Durante M. Clinical indications for carbon ion radiotherapy. Clin Oncol (R Coll Radiol) (2018) 30(5):317–29. doi: 10.1016/j.clon.2018.01.006 29402598

[27] Akamatsu H Karasawa K Omatsu T Isobe Y Ogata R Koba Y. First experience of carbon-ion radiotherapy for early breast cancer. Jpn J Radiol (2014) 32(5):288–95. doi: 10.1007/s11604-014-0300-6 24615166

[28] Hennequin C Barillot I Azria D Belkacemi Y Bollet M Chauvet B. Radiotherapy of breast cancer. Cancer Radiother (2016) 20(Suppl):S139–146. doi: 10.1016/j.canrad.2016.07.025 27522187

[29] Falco M Masojć B Macała A Łukowiak M Woźniak P Malicki J. Deep inspiration breath hold reduces the mean heart dose in left breast cancer radiotherapy. Radiol Oncol (2021) 55(2):212–20. doi: 10.2478/raon-2021-0008 PMC8042816 33600676

[30] Speleers B Schoepen M Belosi F Vakaet V De Neve W Deseyne P. Effects of deep inspiration breath hold on prone photon or proton irradiation of breast and regional lymph nodes. Sci Rep (2021) 11(1):6085. doi: 10.1038/s41598-021-85401-4 33727599 PMC7966795

[31] Verma V Iftekaruddin Z Badar N Hartsell W Han-Chih Chang J Gondi V. Proton beam radiotherapy as part of comprehensive regional nodal irradiation for locally advanced breast cancer. Radiother Oncol (2017) 123(2):294–8. doi: 10.1016/j.radonc.2017.04.007 28457577

[32] Galland-Girodet S Pashtan I MacDonald SM Ancukiewicz M Hirsch AE Kachnic LA. Long-term cosmetic outcomes and toxicities of proton beam therapy compared with photon-based 3-dimensional conformal accelerated partial-breast irradiation: a phase 1 trial. Int J Radiat Oncol Biol Phys (2014) 90(3):493–500. doi: 10.1016/j.ijrobp.2014.04.008 24880212

[33] Thomas A Keller A Menoux I Brahimi Y Vigneron C Le Fèvre C. [Prognostic factors of acute radiodermatitis in breast cancer after adjuvant radiotherapy treated with RT3D or IMRT]. Cancer Radiother (2022) 26(5):684–91. doi: 10.1016/j.canrad.2021.12.004 35227594

[34] Merzenich H Baaken D Schmidt M Bekes I Schwentner L Janni W. Cardiac late effects after modern 3D-conformal radiotherapy in breast cancer patients: a retrospective cohort study in Germany (ESCaRa). Breast Cancer Res Treat (2022) 191(1):147–57. doi: 10.1007/s10549-021-06412-3 PMC8758608 34626275

[35] Beddok A Cottu P Fourquet A Kirova Y. Combination of modern radiotherapy and new targeted treatments for breast cancer management. Cancers (Basel) (2021) 13(24):6358. doi: 10.3390/cancers13246358 34944978 PMC8699586

[36] Darby SC Ewertz M McGale P Bennet AM Blom-Goldman U Bronnum D. Risk of ischemic heart disease in women after radiotherapy for breast cancer. N Engl J Med (2013) 368(11):987–98. doi: 10.1056/NEJMoa1209825 23484825

[37] Choi KH Ahn SJ Jeong JU Yu M Kim JH Jeong BK. Postoperative radiotherapy with intensity-modulated radiation therapy versus 3-dimensional conformal radiotherapy in early breast cancer: A randomized clinical trial of KROG 15-03. Radiother Oncol (2021) 154:179–86. doi: 10.1016/j.radonc.2020.09.043 32980384

[38] Anderson JD Hammond JB Kosiorek HE Thorpe CS Bhangoo RS Pockaj BA. Unplanned implant removal in locally advanced breast cancer. Breast J (2021) 27(5):466–71. doi: 10.1111/tbj.14224 33715231

[39] Karasawa K Omatsu T Arakawa A Yamamoto N Ishikawa T Saito M. A Phase I clinical trial of carbon ion radiotherapy for Stage I breast cancer: clinical and pathological evaluation. J Radiat Res (2019) 60(3):342–7. doi: 10.1093/jrr/rry113 PMC6530622 30805611

[40] Jimenez RB Hickey S DePauw N Yeap BY Batin E Gadd MA. Phase II study of proton beam radiation therapy for patients with breast cancer requiring regional nodal irradiation. J Clin Oncol (2019) 37(30):2778–85. doi: 10.1200/JCO.18.02366 PMC7351324 31449469

[41] Bush DA Do S Lum S Garberoglio C Mirshahidi H Patyal B. Partial breast radiation therapy with proton beam: 5-year results with cosmetic outcomes. Int J Radiat Oncol Biol Phys (2014) 90(3):501–5. doi: 10.1016/j.ijrobp.2014.05.1308 25084608

[42] Chang JH Lee NK Kim JY Kim YJ Moon SH Kim TH. Phase II trial of proton beam accelerated partial breast irradiation in breast cancer. Radiother Oncol (2013) 108(2):209–14. doi: 10.1016/j.radonc.2013.06.008 23891102

[43] Cuaron JJ Chon B Tsai H Goenka A DeBlois D Ho A. Early toxicity in patients treated with postoperative proton therapy for locally advanced breast cancer. Int J Radiat Oncol Biol Phys (2015) 92(2):284–91. doi: 10.1016/j.ijrobp.2015.01.005 PMC4972493 25754632

[44] Pasalic D Strom EA Allen PK Williamson TD Poenisch F Amos RA. Proton accelerated partial breast irradiation: clinical outcomes at a planned interim analysis of a prospective phase 2 trial. Int J Radiat Oncol Biol Phys (2021) 109(2):441–8. doi: 10.1016/j.ijrobp.2020.09.009 32946965

[45] Luo L Cuaron J Braunstein L Gillespie E Kahn A McCormick B. Early outcomes of breast cancer patients treated with post-mastectomy uniform scanning proton therapy. Radiother Oncol (2019) 132:250–6. doi: 10.1016/j.radonc.2018.10.002 30414757

[46] Stick LB Lorenzen EL Yates ES Anandadas C Andersen K Aristei C. Selection criteria for early breast cancer patients in the DBCG proton trial - The randomised phase III trial strategy. Clin Transl Radiat Oncol (2021) 27:126–31. doi: 10.1016/j.ctro.2021.01.012 PMC7892790 33659716

[47] Vaidya JS Bulsara M Wenz F Coombs N Singer J Ebbs S. Reduced mortality with partial-Breast irradiation for early breast cancer: A meta-Analysis of randomized trials. Int J Radiat Oncol Biol Phys (2016) 96(2):259–65. doi: 10.1016/j.ijrobp.2016.05.008 27478165

[48] Bekelman JE Lu H Pugh S Baker K Berg CD Berrington de González A. Pragmatic randomised clinical trial of proton versus photon therapy for patients with non-metastatic breast cancer: the Radiotherapy Comparative Effectiveness (RadComp) Consortium trial protocol. BMJ Open (2019) 9(10):e025556. doi: 10.1136/bmjopen-2018-025556 PMC6797426 31619413

[49] Thorsen LB Offersen BV Danø H Berg M Jensen I Pedersen AN. DBCG-IMN: A population-based cohort study on the effect of internal mammary node irradiation in early node-positive breast cancer. J Clin Oncol (2016) 34(4):314–20. doi: 10.1200/JCO.2015.63.6456 26598752

[50] Grau C Durante M Georg D Langendijk JA Weber DC. Particle therapy in europe. Mol Oncol (2020) 14(7):1492–9. doi: 10.1002/1878-0261.12677 PMC7332216 32223048

[51] Durante M Parodi K. Radioactive beams in particle therapy: past, present, and future. Front Phys (2020) 8:00326. doi: 10.3389/fphy.2020.00326 33224941 PMC7116396

[52] Ricardi U Maraldo MV Levis M Parikh RR. Proton therapy for lymphomas: current state of the art. Onco Targets Ther (2019) 12:8033–46. doi: 10.2147/OTT.S220730 PMC6781741 31632057

[53] Iorio GC Salvestrini V Borghetti P De Felice F Greco C Nardone V. The impact of modern radiotherapy on radiation-induced late sequelae: Focus on early-stage mediastinal classical Hodgkin Lymphoma. A critical review by the Young Group of the Italian Association of Radiotherapy and Clinical Oncology (AIRO). Crit Rev Oncol Hematol (2021) 161:103326. doi: 10.1016/j.critrevonc.2021.103326 33862247

[54] Park JM Kim JI Wu HG. Technological advances in charged-particle therapy. Cancer Res Treat (2021) 53(3):635–40. doi: 10.4143/crt.2021.706 PMC8291177 34176252

[55] Giuliano AE Ballman KV McCall L Beitsch PD Brennan MB Kelemen PR. Effect of axillary dissection vs no axillary dissection on 10-Year overall survival among women with invasive breast cancer and sentinel node metastasis: the ACOSOG Z0011 (Alliance) randomized clinical trial. JAMA (2017) 318(10):918–26. doi: 10.1001/jama.2017.11470 PMC5672806 28898379

[56] Poortmans PM Weltens C Fortpied C Kirkove C Peignaux-Casasnovas K Budach V. Internal mammary and medial supraclavicular lymph node chain irradiation in stage I-III breast cancer (EORTC 22922/10925): 15-year results of a randomised, phase 3 trial. Lancet Oncol (2020) 21(12):1602–10. doi: 10.1016/S1470-2045(20)30472-1 33152277

[57] Kowalchuk RO Corbin KS Jimenez RB. Particle therapy for breast cancer. Cancers (Basel) (2022) 14(4):189–206. doi: 10.3390/cancers14041066 PMC8870138 35205814

[58] Malouff TD Mahajan A Mutter RW Krishnan S Hoppe BS Beltran C. Carbon ion radiation therapy in breast cancer: a new frontier. Breast Cancer Res Treat (2020) 181(2):291–6. doi: 10.1007/s10549-020-05641-2 PMC7734650 32318954

[59] Heymann S Dipasquale G Nguyen NP San M Gorobets O Leduc N. Two-Level factorial pre-TomoBreast pilot study of tomotherapy and conventional radiotherapy in breast cancer: *post hoc* utility of a mean absolute dose deviation penalty score. Technol Cancer Res Treat (2020) 19:1533033820947759. doi: 10.1177/1533033820947759 32940569 PMC7502852

[60] Piruzan E Vosoughi N Mahdavi SR Khalafi L Mahani H. Target motion management in breast cancer radiation therapy. Radiol Oncol (2021) 55(4):393–408. doi: 10.2478/raon-2021-0040 34626533 PMC8647788

[61] Fuchs H Elia A Resch AF Kuess P Luhr A Vidal M. Computer-assisted beam modeling for particle therapy. Med Phys (2021) 48(2):841–51. doi: 10.1002/mp.14647 PMC7986420 33283910

[62] Byun HK Han MC Yang K Kim JS Yoo GS Koom WS. Physical and biological characteristics of particle therapy for oncologists. Cancer Res Treat (2021) 53(3):611–20. doi: 10.4143/crt.2021.066 PMC8291193 34139805

[63] Paganetti H. Relating proton treatments to photon treatments *via* the relative biological effectiveness-should we revise current clinical practice? Int J Radiat Oncol Biol Phys (2015) 91(5):892–4. doi: 10.1016/j.ijrobp.2014.11.021 25832682

[64] Cuaron JJ Chang C Lovelock M Higginson DS Mah D Cahlon O. Exponential increase in relative biological effectiveness along distal edge of a proton bragg peak as measured by deoxyribonucleic acid double-strand breaks. Int J Radiat Oncol Biol Phys (2016) 95(1):62–9. doi: 10.1016/j.ijrobp.2016.02.018 PMC5005074 27084629

[65] Hwang EJ Gorayski P Le H Hanna GG Kenny L Penniment M. Particle therapy tumour outcomes: An updated systematic review. J Med Imaging Radiat Oncol (2020) 64(5):711–24. doi: 10.1111/1754-9485.13021 32270626

[66] Geyer CE Jr. Bandos H Rastogi P Jacobs SA Robidoux A Fehrenbacher L. Correction to: Definitive results of a phase III adjuvant trial comparing six cycles of FEC-100 to four cycles of AC in women with operable node-negative breast cancer: the NSABP B-36 trial (NRG Oncology). Breast Cancer Res Treat (2022) 193(3):565. doi: 10.21203/rs.3.rs-841103/v1 35507135 PMC9235403

[67] Gelber RD Wang XV Cole BF Cameron D Cardoso F Tjan-Heijnen V. Six-year absolute invasive disease-free survival benefit of adding adjuvant pertuzumab to trastuzumab and chemotherapy for patients with early HER2-positive breast cancer: A Subpopulation Treatment Effect Pattern Plot (STEPP) analysis of the APHINITY (BIG 4-11) trial. Eur J Cancer (2022) 166:219–28. doi: 10.1016/j.ejca.2022.01.031 35313167

[68] de Boo LW Jóźwiak K Joensuu H Lindman H Lauttia S Opdam M. Adjuvant capecitabine-containing chemotherapy benefit and homologous recombination deficiency in early-stage triple-negative breast cancer patients. Br J Cancer (2022) 126(10):1401–9. doi: 10.1038/s41416-022-01711-y PMC9090783 35124703

[69] Mayer EL Fesl C Hlauschek D Garcia-Estevez L Burstein HJ Zdenkowski N. Treatment exposure and discontinuation in the PALbociclib coLlaborative adjuvant study of palbociclib with adjuvant endocrine therapy for hormone receptor-Positive/Human epidermal growth factor receptor 2-Negative early breast cancer (PALLAS/AFT-05/ABCSG-42/BIG-14-03). J Clin Oncol (2022) 40(5):449–58. doi: 10.1200/JCO.21.01918 PMC9851679 34995105

[70] Fu Z Lin Z Yang M Li C. Cardiac toxicity from adjuvant targeting treatment for breast cancer post-surgery. Front Oncol (2022) 12:706861. doi: 10.3389/fonc.2022.706861 35402243 PMC8988147

[71] Hackshaw MD Danysh HE Singh J Ritchey ME Ladner A Taitt C. Incidence of pneumonitis/interstitial lung disease induced by HER2-targeting therapy for HER2-positive metastatic breast cancer. Breast Cancer Res Treat (2020) 183(1):23–39. doi: 10.1007/s10549-020-05754-8 32591987 PMC7376509

[72] Luo W Ali YF Liu C Wang Y Liu C Jin X. Particle therapy for breast cancer: benefits and challenges. Front Oncol (2021) 11:662826. doi: 10.3389/fonc.2021.662826 34026640 PMC8131859

[73] Elmore LC Li M Lin H Shen Y Shaitelman SF Babiera G. Impact of medicaid expansion under the affordable care act on receipt of surgery for breast cancer. Ann Surg Open (2022) 3(3):e194. doi: 10.1097/AS9.0000000000000194 36199482 PMC9508982

[74] Pilewskie ML Dimick JB. Bariatric surgery for breast cancer risk reduction-benefit may not be one size fits all. JAMA Surg (2023) 158(6):641–2. doi: 10.1001/jamasurg.2023.0534 37043229

[75] van de Voort EMF Franckena M Struik GM Moelker A Klem T. Comment on: cryoablation without excision for low-risk early-stage breast cancer: 3-year interim analysis of ipsilateral breast tumor recurrence in the ICE3 trial. Ann Surg Oncol (2023) 30(6):3284–5. doi: 10.1245/s10434-023-13335-4 37036591

